# Emerging foodborne diseases: an evolving public health challenge.

**DOI:** 10.3201/eid0304.970403

**Published:** 1997

**Authors:** R V Tauxe

**Affiliations:** Centers for Disease Control and Prevention, Atlanta, Georgia 30333, USA.

## Abstract

The epidemiology of foodborne disease is changing. New pathogens have emerged, and some have spread worldwide. Many, including Salmonella, Escherichia coli O157:H7, Campylobacter, and Yersinia enterocolitica, have reservoirs in healthy food animals, from which they spread to an increasing variety of foods. These pathogens cause millions of cases of sporadic illness and chronic complications, as well as large and challenging outbreaks over many states and nations. Improved surveillance that combines rapid subtyping methods, cluster identification, and collaborative epidemiologic investigation can identify and halt large, dispersed outbreaks. Outbreak investigations and case-control studies of sporadic cases can identify sources of infection and guide the development of specific prevention strategies. Better understanding of how pathogens persist in animal reservoirs is also critical to successful long-term prevention. In the past, the central challenge of foodborne disease lay in preventing the contamination of human food with sewage or animal manure. In the future, prevention of foodborne disease will increasingly depend on controlling contamination of feed and water consumed by the animals themselves.


### Emerging Foodborne Diseases: An Evolving Public Health Challenge

**Robert V. Tauxe**
Centers for Disease Control and Prevention, Atlanta, Georgia, USA

The epidemiology of foodborne disease is changing. New pathogens have emerged, and some have spread worldwide. Many, including *Salmonella*, *Escherichia coli* O157:H7, *Campylobacter*, and *Yersinia enterocolitica*, have reservoirs in healthy food animals, from which they spread to an increasing variety of foods. These pathogens cause millions of cases of sporadic illness and chronic complications, as well as large and challenging outbreaks over many states and nations. Improved surveillance that combines rapid subtyping methods, cluster identification, and collaborative epidemiologic investigation can identify and halt large, dispersed outbreaks. Outbreak investigations and case-control studies of sporadic cases can identify sources of infection and guide the development of specific prevention strategies. Better understanding of how pathogens persist in animal reservoirs is also critical to successful long-term prevention. In the past, the central challenge of foodborne disease lay in preventing the contamination of human food with sewage or animal manure. In the future, prevention of foodborne disease will increasingly depend on controlling contamination of feed and water consumed by the animals themselves.

Every year, in the United States foodborne infections cause millions of illnesses and thousands of deaths; most infections go undiagnosed and unreported. As the epidemiology of foodborne infections evolves, old scenarios and solutions need to be updated. This article reviews main trends in the evolution of foodborne disease epidemiology and their effect on surveillance and prevention activities.

Preventing foodborne disease is a multifaceted process, without simple and universal solutions. For most foodborne pathogens, no vaccines are available. Consumer education about basic principles of food safety, an important component of prevention, by itself is insufficient. Food reaches the consumer through long chains of industrial production, in which many opportunities for contamination exist. The general strategy of prevention is to understand the mechanisms by which contamination and disease transmission can occur well enough to interrupt them. An outbreak investigation or epidemiologic study should go beyond identifying a suspected food and pulling it from the shelf to defining the chain of events that allowed contamination with an organism in large enough numbers to cause illness. We learn from the investigation what went wrong, in order to devise strategies to prevent similar events in the future. Although outbreaks make the news, most foodborne infections occur as individual or sporadic cases. Therefore, the sources of sporadic cases must also be investigated and understood.

Address for correspondence: Robert V. Tauxe, Centers for Disease Control and Prevention, 1600 Clifton Road, MS A38, Atlanta, GA 30333 USA; fax: 404-639-2205.

### Emerging Foodborne Pathogens

Substantial progress has been made in preventing foodborne diseases. For example, typhoid fever, extremely common at the beginning of the 20th century, is now almost forgotten in the United States. It was conquered in the preantibiotic era by disinfection of drinking water, sewage treatment, milk sanitation and pasteurization, and shellfish bed sanitation (Figure 1). Similarly, cholera, bovine tuberculosis, and trichinosis have also been controlled in the United States. However, new foodborne pathogens have emerged. Among the first of these were infections caused by nontyphoid strains of *Salmonella*, which have increased decade by decade since World War II (Figure 1).

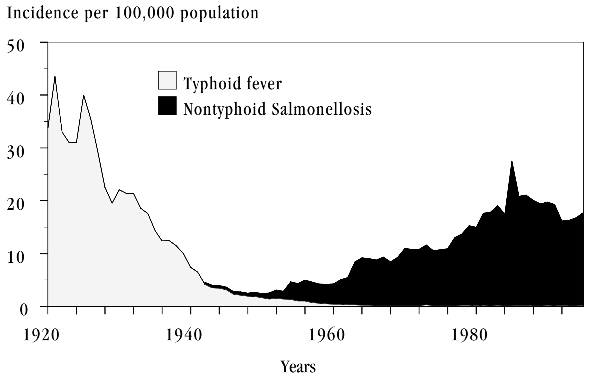

Figure 1. Reported incidence of typhoid fever and nontyphoidal salmonellosis in the United States, 1920–1995.

Table 1. New pathogens that are foodborne and pathogens newly recognized as predominantly foodborne in the United States in the last 20 years

| |
|---|
| *Campylobacter jejuni* |
| *Campylobacter fetus* ssp. *fetus* |
| *Cryptosporidium cayetanensis* |
| *Escherichia coli* O157:H7 and related *E. coli* (e.g., O111:NM, O104:H21) |
| *Listeria monocytogenes* |
| Norwalk-like viruses |
| *Nitzschia pungens* (cause of amnesic shellfish poisoning) |
| *Salmonella* Enteritidis |
| *Salmonella* Typhimurium DT 104 |
| *Vibrio cholerae* O1 |
| *Vibrio vulnificus* |
| *Vibrio parahaemolyticus* |
| *Yersinia enterocolitica* |

In the last 20 years, other infectious agents have been either newly described or newly associated with foodborne transmission (Table 1). *Vibrio vulnificus*, *Escherichia coli* O157:H7, and *Cyclospora cayetanensis* are examples of newly described pathogens that often are foodborne. *V. vulnificus* was identified in the bloodstream of persons with underlying liver disease who had fulminant infections after eating raw oysters or being exposed to seawater; this organism lives in the sea and can be a natural summertime commensal organism in shellfish (1). *E. coli* O157:H7 was first identified as a pathogen in 1982 in an outbreak of bloody diarrhea traced to hamburgers from a fast-food chain (2); it was subsequently shown to have a reservoir in healthy cattle (3). *Cyclospora,* known previously as a cyanobacterialike organism, received its current taxonomic designation in 1992 and emerged as a foodborne pathogen in outbreaks traced to imported Guatemalan raspberries in 1996 (4,5). The similarity of *Cyclospora* to *Eimeria* coccidian pathogens of birds suggests an avian reservoir (4,5).

Some known pathogens have only recently been shown to be predominantly foodborne. For example, *Listeria monocytogenes* was long known as a cause of meningitis and other invasive infections in immunocompromised hosts. How these hosts became infected remained unknown until a series of investigations identified food as the most common source (6). Similarly, *Campylobacter jejuni* was known as a rare opportunistic bloodstream infection until veterinary diagnostic methods used on specimens from humans showed it was a common cause of diarrheal illness (7). Subsequent epidemiologic investigations implicated poultry and raw milk as the most common sources of sporadic cases and outbreaks, respectively (8). *Yersinia enterocolitica,* rare in the United States but a common cause of diarrheal illness and pseudoappendicitis in northern Europe and elsewhere, is now known to be most frequently associated with undercooked pork (9).

These foodborne pathogens share a number of characteristics. Virtually all have an animal reservoir from which they spread to humans; that is, they are foodborne zoonoses. In marked contrast to many established zoonoses, these new zoonoses do not often cause illness in the infected host animal. The chicken with lifelong ovarian infection with *Salmonella* serotype Enteritidis, the calf carrying *E. coli* O157:H7, and the oyster carrying Norwalk virus or *V. vulnificus* appear healthy; therefore, public health concerns must now include apparently healthy animals. Limited existing research on how animals acquire and transmit emerging pathogens among themselves often implicates contaminated fodder and water; therefore, public health concerns must now include the safety of what food animals themselves eat and drink.

For reasons that remain unclear, these pathogens can rapidly spread globally. For example, *Y. enterocolitica* spread globally among pigs in the 1970s (10); *Salmonella* serotype Enteritidis appeared simultaneously around the world in the 1980s (11); and *Salmonella* Typhimurium Definitive Type (DT) 104 is now appearing in North America, Europe, and perhaps elsewhere (12); therefore, public health concerns must now include events happening around the world, as harbingers of what may appear here.

Many emerging zoonotic pathogens are becoming increasingly resistant to antimicrobial agents, largely because of the widespread use of antibiotics in the animal reservoir. For example, *Campylobacter* isolated from human patients in Europe is now increasingly resistant to fluoroquinolones, after these agents were introduced for use in animals (13). Salmonellae have become increasing resistant to a variety of antimicrobial agents in the United States (14); therefore, public health concerns must include the patterns of antimicrobial use in agriculture as well as in human medicine.

The foods contaminated with emerging pathogens usually look, smell, and taste normal, and the pathogen often survives traditional preparation techniques: *E. coli* O157:H7 in meat can survive the gentle heating that a rare hamburger gets (15); *Salmonella* Enteritidis in eggs survives in an omelette (16); and Norwalk virus in oysters survives gentle steaming (17). Following standard and traditional recipes can cause illness and outbreaks. Contamination with the new foodborne zoonoses eludes traditional food inspection, which relies on visual identification of foodborne hazards. These pathogens demand new control strategies, which would minimize the likelihood of contamination in the first place. The rate at which new pathogens have been identified suggests that many more remain to be discovered. Many of the foodborne infections of the future are likely to arise from the animal reservoirs from which we draw our food supply.

Once a new foodborne disease is identified, a number of critical questions need to be answered to develop a rational approach to prevention: What is the nature of the disease? What is the nature of the pathogen? What are simple ways to easily identify the pathogen and diagnose the disease? What is the incidence of the infection? How can the disease be treated? Which foods transmit the infection? How does the pathogen get into the food, and how well does it persist there? Is there is an animal reservoir? How do the animals themselves become infected? How can the disease be prevented? Does the prevention strategy work?

The answers to these questions do not come rapidly. Knowledge accumulates gradually, as a result of detailed scientific investigations, often conducted during outbreaks (18). After 15 years of research, we know a great deal about infections with *E. coli* O157:H7, but we still do not know how best to treat the infection, nor how the cattle (the principal source of infection for humans) themselves become infected. Better slaughter procedures and pasteurization of milk are useful control strategies for this pathogen in meat and milk, as irradiation of meat may be in the future. More needs to be learned: for example, it remains unclear how best to prevent this organism from contaminating lettuce or apple juice. For more recently identified agents, even less is known.

### New Food Vehicles of Transmission

Along with new pathogens, an array of new food vehicles of transmission have been implicated in recent years. Traditionally, the food implicated in a foodborne outbreak was undercooked meat, poultry or seafood, or unpasteurized milk. Now, additional foods previously thought safe are considered hazardous. For example, for centuries, the internal contents of an egg were presumed safe to eat raw. However, epidemic *Salmonella* Enteritidis infection among egg-laying flocks indicates that intact eggs may have internal contamination with this *Salmonella* serotype. Many outbreaks are caused by contaminated shell eggs, including eggs used in such traditional recipes as eggnog and Caesar salad, lightly cooked eggs in omelettes and French toast, and even foods one would presume thoroughly cooked, such as lasagna and meringue pie (19,20). *E. coli* O157:H7 has caused illness through an ever-broadening spectrum of foods, beyond the beef and raw milk that are directly related to the bovine reservoir. In 1992, an outbreak caused by apple cider showed that this organism could be transmitted through a food with a pH level of less than 4.0, possibly after contact of fresh produce with manure (21). A recent outbreak traced to venison jerky suggests a wild deer reservoir, so both cattle and feral deer manure are of concern (22). Imported raspberries contaminated with *Cyclospora* caused an epidemic in the United States in 1996, possibly because contaminated surface water was used to spray the berries with fungicide before harvest (5). Norwalk-like viruses, which appear to have a human reservoir, have contaminated oysters harvested from pristine waters by oyster catchers who did not use toilets with holding tanks on their boats and were themselves the likely source of the virus (23).

The new food vehicles of disease share several features. Contamination typically occurs early in the production process, rather than just before consumption. Because of consumer demand and the global food market, ingredients from many countries may be combined in a single dish, which makes the specific source of contamination difficult to trace. These foods have fewer barriers to microbial growth, such as salt, sugar, or preservatives; therefore, simple transgressions can make the food unsafe. Because the food has a short shelf life, it may often be gone by the time the outbreak is recognized; therefore, efforts to prevent contamination at the source are very important.

An increasing, though still limited, proportion of reported foodborne outbreaks are being traced to fresh produce (24). A series of outbreaks recently investigated by the Centers for Disease Control and Prevention (CDC) has linked a variety of pathogens to fresh fruits and vegetables harvested in the United States and elsewhere (Table 2). The investigations have often been triggered by detection of more cases than expected of a rare serotype of *Salmonella* or *Shigella* or by diagnosis of a rare infection like cyclosporiasis. Outbreaks caused by common serotypes are more likely to be missed. Various possible points of contamination have been identified during these investigations, including contamination during production and harvest, initial processing and packing, distribution, and final processing (Table 3). For example, fresh or inadequately composted manure is used sometimes, although *E. coli* O157:H7 has been shown to survive for up to 70 days in bovine feces (25). Untreated or contaminated water seems to be a particularly likely source of contamination. Water used for spraying, washing, and maintaining the appearance of produce must be microbiologically safe. After two large outbreaks of salmonellosis were traced to imported cantaloupe, the melon industry considered a "Melon Safety Plan," focusing particularly on the chlorination of water used to wash melons and to make ice for shipping them. Although the extent to which the plan was implemented is unknown, no further large outbreaks have occurred. After two large outbreaks of salmonellosis were traced to a single tomato packer in the Southeast, an automated chlorination system was developed for the packing plant wash tank. Because tomatoes absorb water (and associated bacteria) if washed in water colder than they are, particular attention was also focused on the temperature of the water bath (26,27). No further outbreaks have been linked to southeastern tomatoes. Similar attention is warranted for water used to rinse lettuce heads in packing sheds and to crisp them in grocery stores as well as for water used in processing other fresh produce.

Table 2. Foodborne outbreaks traced to fresh produce, 1990–1996

| Yr. | Pathogen | Vehicle | Cases (No.) | States (No.) | Source |
|---|---|---|---|---|---|
| '90 | *S.* Chester | Cantaloupe | 245 | 30 | C.A.[a] |
| '90 | *S.* Javiana | Tomatoes | 174 | 4 | U.S.[b] |
| '90 | Hepatitis A | Strawberries | 18 | 2 | U.S. |
| '91 | *S.* Poona | Cantaloupe | >400 | 23 | U.S./ C.A. |
| '93 | *E. coli* O157:H7 | Apple cider | 23 | 1 | U.S. |
| '93 | *S.* Montevideo | Tomatoes | 84 | 3 | U.S. |
| '94 | *Shigella flexneri* | Scallions | 72 | 2 | C.A. |
| '95 | *S.* Stanley | Alfalfa sprouts | 242 | 17 | N.K.[c] |
| '95 | *S.* Hartford | Orange juice | 63 | 21 | U.S. |
| '95 | *E. coli* O157:H7 | Leaf lettuce | 70 | 1 | U.S. |
| '96 | *E. coli* O157:H7 | Leaf lettuce | 49 | 2 | U.S. |
| '96 | Cyclospora | Raspberries | 978 | 20 | C.A. |
| '96 | *E. coli* O157:H7 | Apple juice | 71 | 3 | U.S. |

[a]Central America
[b]United States
[c]Source not known

Table 3. Events and potential contamination sources during produce processing

| Event | Contamination sources |
|---|---|
| Production and harvest | |
| Growing, picking, bundling | Irrigation water, manure, lack of field sanitation |
| Initial processing | |
| Washing, waxing, sorting, boxing | Wash water, handling |
| Distribution | |
| Trucking | Ice, dirty trucks |
| Final processing | |
| Slicing, squeezing, shredding, peeling | Wash water, handling, cross-contamination |

### A New Outbreak Scenario

Because of changes in the way food is produced and distributed, a new kind of outbreak has appeared. The traditional foodborne outbreak scenario often follows a church supper, family picnic, wedding reception, or other social event. This scenario involves an acute and highly local outbreak, with a high inoculum dose and a high attack rate. The outbreak is typically immediately apparent to those in the local group, who promptly involve medical and public health authorities. The investigation identifies a food-handling error in a small kitchen that occurs shortly before consumption. The solution is also local. Such outbreaks still occur, and handling them remains an important function of a local health department.

However, diffuse and widespread outbreaks, involving many counties, states, and even nations (28), are identified more frequently and follow an entirely different scenario. The new scenario is the result of low-level contamination of a widely distributed commercial food product. In most jurisdictions, the increase in cases may be inapparent against the background illness. The outbreak is detected only because of a fortuitous concentration of cases in one location, because the pathogen causing the outbreak is unusual, or because laboratory-based subtyping of strains collected over a wide area identifies a diffuse surge in one subtype. In such outbreaks, investigation can require coordinated efforts of a large team to clarify the extent of the outbreak, implicate a specific food, and determine the source of contamination. Often, no obvious terminal food-handling error is found. Instead, contamination is the result of an event in the industrial chain of food production. Investigating, controlling, and preventing such outbreaks can have industrywide implications.

These diffuse outbreaks can be caused by a variety of foods. Because fresh produce is usually widely distributed, most of the produce-related outbreaks listed in Table 2 were multistate events. Some of the largest outbreaks affected most states at once. For example, a recent outbreak of *Salmonella* Enteritidis infections caused by a nationally distributed brand of ice cream affected the entire nation (29). Although it caused an estimated 250,000 illnesses, it was detected only when vigorous routine surveillance identified a surge in reported infections with *S.* Enteritidis in one area of southern Minnesota. The consumers affected did not make food-handling errors with their ice cream, so food safety instruction could not have prevented this outbreak. The ice cream premix was transported after pasteurization to the ice cream factory in tanker trucks that had been used to haul raw eggs. The huge epidemic was the result of a basic failure on an industrial scale to separate the raw from the cooked.

*S.* Enteritidis infections also illustrate why surveillance and investigation of sporadic cases are needed. A diffuse increase in sporadic cases can occur well before a local or large outbreak focuses attention on the emergence of a pathogen. The isolation rate for *S.* Enteritidis began to increase sharply in the New England region in 1978 (Figure 2); all cases were sporadic. In 1982, an outbreak in a New England nursing home was traced to eggs from a local supplier. However, the egg connection was not really appreciated until 1986, when a large multistate outbreak of *S.* Enteritidis infections was traced to stuffed pasta made with raw eggs and labeled “fully cooked.” This outbreak, affecting an estimated 3,000 persons in seven states, led to the documentation that *S.* Enteritidis was present on egg-laying farms and to the subsequent demonstration that both outbreaks and sporadic cases of infections were associated with shell eggs (19,30). Since then, Enteritidis has become the most common serotype of *Salmonella* isolated in the United States, accounting for 25% of all *Salmonella* reported in the country and causing outbreaks coast to coast. Eggs remain the dominant source of these infections, causing large outbreaks when they are pooled and undercooked and individual sporadic cases among consumers who eat individual eggs (20,31). Perhaps focused investigation and control measures taken when the localized increase in sporadic *Salmonella* cases was just beginning might have prevented the subsequent spread.

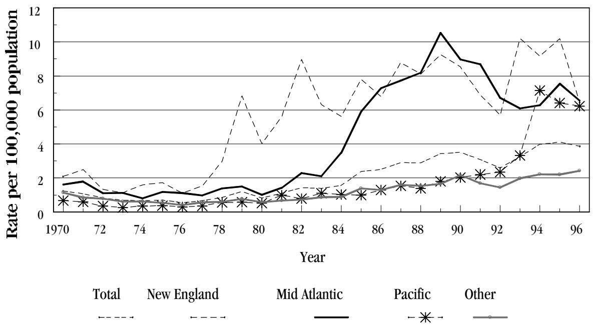

Figure 2. *Salmonella* Enteritidis isolation rates from humans by region, United States, 1970–1996.

### Changing Surveillance Strategies

In the United States, surveillance for diseases of major public health importance has been conducted for many years. The legal framework for surveillance resides in the state public health epidemiology offices, which share data with CDC. The first surveillance systems depended on physician or coroner notification of specific diseases and conditions, with reports going first to the local health department, then to state and federal offices. Now electronic, this form of surveillance is still used for many specific conditions (32). In 1962, a second channel was developed specifically for *Salmonella*, to take advantage of the added public health information provided by subtyping the strains of bacteria (33). Clinical laboratories that isolated *Salmonella* from humans were requested or required to send the strains to the state public health laboratory for serotyping. Although knowing the serotype is usually of little benefit to the individual patient, it has been critical to protecting and improving the health of the public at large. Serotyping allows cases that might otherwise appear unrelated to be included in an investigation because they are of the same serotype. Moreover, infections that are close in time and space to an outbreak but are caused by nonoutbreak serotypes and are probably unrelated can be discounted. Results of serotyping are now sent electronically from public health laboratories and can be rapidly analyzed and summarized. *Salmonella* serotyping was the first subtype-based surveillance system and is a model for similar systems (34). Yet another source of surveillance data involves summary reports of foodborne disease outbreak investigations from local and state health departments (35). About 400 such outbreaks are reported annually, by a system that remains paper-based, labor-intensive, and slow.

Existing surveillance systems provide a limited and relatively inexpensive net for tracing large-scale trends in foodborne diseases under surveillance and for detecting outbreaks of established pathogens in the United States. However, they are less sensitive to diffuse outbreaks of common pathogens, provide little detail on sporadic cases, and are not easy to extend to emerging pathogens. In the future, changes in health delivery may impinge on the way that diagnoses are made and reported, leading to artifactual changes in reported disease incidence.

Therefore, CDC, in collaboration with state health departments and federal food regulatory agencies, is enhancing national surveillance for foodborne diseases in several ways. First, the role of subtyping in public health laboratories is being expanded to encompass new molecular subtyping methods. Beginning in 1997, a national subtyping network for *E. coli* O157:H7 of participating state public health department laboratories and CDC will use a single standardized laboratory protocol to subtype strains of this important pathogen. The standard method, pulsed-field gel electrophoresis, can be easily adapted to other bacterial pathogens. In this network, each participating laboratory will be able to routinely compare the genetic gel patterns of strains of *E. coli* O157:H7 with the patterns in a national pattern bank. This will enable rapid detection of clusters of related cases within the state and will focus investigative resources on the cases most likely to be linked. It will also enable related cases scattered across several states to be linked so that a common source can be sought.

Another surveillance strategy, now implemented, is active surveillance in sentinel populations. Since January 1996, at five U.S. sentinel sites, additional surveillance resources make it possible to contact laboratories directly for regular reporting of bacterial infections likely to be foodborne (36; Figure 3). In addition, surveys of the population, physicians, and laboratories measure the proportion of diarrheal diseases that are undiagnosed and unreported so that the true disease incidence can be estimated. This surveillance, known as FoodNet, is the platform on which more detailed investigations, including case-control studies of sporadic cases of common foodborne infections, are being conducted.

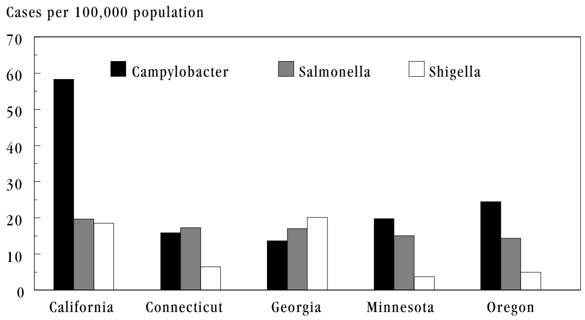

Figure 3. Incidence of three infections in FoodNet surveillance areas, 1996.

Yet another new surveillance initiative is the routine monitoring of antimicrobial resistance among a sample of *Salmonella* and *E. coli* O157:H7 bacteria isolated from humans (37). A new cluster detection algorithm is being applied routinely to surveillance data for *Salmonella* at the national level, making it possible to detect and flag possible outbreaks as soon as the data are reported (38). Implementation of such algorithms for other infections and at the state level will further increase the usefulness of routine surveillance.

Further enhancements are possible as active surveillance through FoodNet is extended to a wider spectrum of infections, including foodborne parasitic and viral infections. In 1997, active surveillance for *Cyclospora* began in FoodNet, which quickly resulted in the detection of a diffuse outbreak among persons who had been on a Caribbean cruise ship that made stops in Mexico and Central America (CDC, unpub. data). Application of standardized molecular subtyping methods to other foodborne pathogens will provide a more sensitive warning system for diffuse outbreaks of a variety of pathogens. To handle outbreaks in areas not covered by FoodNet, standard surveillance and investigative capacities in state health department epidemiology offices and laboratories should be strengthened. In addition, enhanced international consultation will be critical to better detect and investigate international or global outbreaks (28).

### Implications of the New Outbreak Scenario for Public Health Activities

Our public health infrastructure is tiered, both in surveillance responsibilities and in response to emergency situations (39). At the local level, the county or city health department, first developed in response to epidemic cholera and other challenges in the 19th century, is responsible for most basic surveillance, investigation, and prevention activities. At the state level, epidemiologists, public health laboratorians, sanitarians, and educators conduct statewide surveillance and prevention activities and consult with and support local authorities. At the national level, CDC is the primary risk-assessment agency for public health hazards and conducts the primary national surveillance as well as epidemic response in support of state health departments. The Food and Drug Administration, Department of Agriculture, and Environmental Protection Agency are the primary regulatory agencies, charged with specific responsibilities regarding the nation's food and water supplies that interlock and are not always predictable. The Food and Drug Administration regulates low-acid canned foods, imported foods, pasteurized milk, many seafoods, rabbits raised for meat, and food and water provided on aircraft and trains. The Department of Agriculture regulates meat and poultry, including primary slaughter and further processing, and pasteurized eggs; investigates animal and plant diseases; and maintains the county extension outreach program. Shell eggs do not have a clear regulatory home, as the Department of Agriculture regulates the grading of shell eggs for quality, but the Food and Drug Administration, since 1995, has responsibility for the microbiologic safety of shell eggs.

The new outbreak scenario has several implications for the practice of public health, starting at the local level. One is that when diffuse outbreaks are detected, a local health department may need to investigate a few cases that are part of a larger outbreak despite their apparently small local impact. Second, an apparently local outbreak may herald the first recognized manifestation of a national or even international event.

When a diffuse outbreak of a potentially foodborne pathogen is detected, rapid investigation is needed to determine whether the outbreak is foodborne, and if possible, identify a specific food vehicle. These investigations, which typically include case-control studies, may need to be conducted in several locations at once. While all cases or all affected states may not need to be included in such an investigation, combining cases from several locations in one investigation and repeating the investigation in more than one location can be helpful. For example, in a recent international outbreak of *Salmonella* Stanley infections traced to alfalfa sprouts, concentrations of cases in Arizona, Michigan, and Finland led to case-control studies in each location, each of which linked illness to eating sprouts grown from the same batch of alfalfa seeds. This proved that the seeds were contaminated at the source (40). Parallel investigations can also lead to new twists. In the large West Coast outbreak of *E. coli* O157:H7 infections in 1993, a parallel investigation conducted in Nevada identified a type of hamburger other than the one implicated in the initial case-control investigation in Washington, leading to a broader recall and a more complete investigation of the circumstances of contamination (15,41). Because well-conducted investigations may lead to major product recalls, industrial review, and overhaul, and even international embargoes, it is essential that they be of the highest scientific quality.

Foodborne outbreaks are investigated for two main reasons. The first is to identify and control an ongoing source by emergency action: product recall, restaurant closure, or other temporary but definitive solutions. The second reason is to learn how to prevent future similar outbreaks from occurring. In the long run this second purpose will have an even greater impact on public health than simply identifying and halting the outbreaks. Because all the answers are not available and existing regulations may not be sufficient to prevent outbreaks, the scientific investigation often requires a careful evaluation of the chain of production. This traceback is an integral part of the outbreak investigation. It is not a search for regulatory violations, but rather an effort to determine where and how contamination occurred. Often, the contamination scenario reveals that a critical point has been lost. Therefore, epidemiologists must participate in traceback investigations.

Intervention during outbreaks often depends on having enough good epidemiologic data to act with confidence, without waiting for a definitive laboratory test, particularly if potentially lethal illnesses are involved. For example, if five persons with classic clinical botulism ate at the same restaurant the preceding day (but have nothing else apparent in common), prudence dictates closing the restaurant quickly while the outbreak is sorted out—that is, before a specific food is identified or confirmatory cultures are made, which may take several days or even weeks. Good epidemiologic data, including evidence of a clear statistical association with a specific exposure, biologic plausibility of the illness syndrome, the potential hazard of that food, and the logical consistency of distribution of the suspect food and cases are essential.

The role of the regulatory agency laboratory is also affected by the new scenario. Because of the short shelf life and broad distribution of many of the new foods responsible for infection, by the time the outbreak is recognized and investigated the relevant food may no longer be available for culture. Because contamination may be restricted to a single production lot, blind sampling of similar foods that does not include the implicated lot can give a false sense of security. Good epidemiologic information pointing to contamination of a specific food or production lot should guide the microbiologic sampling and the interpretation of the results. Available methods may be insufficient to detect low-level contamination, even of well-established pathogens.

### New Approaches to the Prevention of Foodborne Disease

Meeting the complex challenge of foodborne disease prevention will require the collaboration of regulatory agencies and industry to make food safely and keep it safe throughout the industrial chain of production. Prevention can be “built in” to the industry by identifying and controlling the key points—from field, farm, or fishing ground to the dinner table—at which contamination can either occur or be eliminated. The general strategy known as Hazard Analysis and Critical Control Points (HACCP) replaces the strategy of final product inspection. Some simple control strategies are self-evident, once the reality of microbial contamination is recognized. For example, shipping fruit from Central America with clean ice or in closed refrigerator trucks, rather than with ice made from untreated river water, is common sense. Similarly, requiring oyster harvesters to use toilets with holding tanks on their oyster boats is an obvious way to reduce fecal contamination of shallow oyster beds. Pasteurization provides the extra barrier that will prevent *E. coli* O157:H7 and other pathogens from contaminating a large batch of freshly squeezed juice.

For many foodborne diseases, multiple choices for prevention are available, and the best answer may be to apply several steps simultaneously. For *E. coli* O157:H7 infections related to the cattle reservoir, pasteurizing milk and cooking meat thoroughly provide an important measure of protection but are insufficient by themselves. Options for better control include continued improvements in slaughter plant hygiene and control measures under HACCP, developing additives to cattle feed that alter the microbial growth either in the feed or in the bovine rumen to make cows less hospitable hosts for *E. coli* O157, immunizing or otherwise protecting the cows so that they do not become infected in the first place, and irradiating beef after slaughter. For *C. jejuni* infections related to the poultry reservoir, future control options may include modification of the slaughter process to reduce contamination of chicken carcasses by bile or by water baths, freezing chicken carcasses to reduce *Campylobacter* counts, chlorinating the water that chickens drink to prevent them from getting infected, vaccinating chickens, and irradiating poultry carcasses after slaughter.

Outbreaks are often fertile sources of new research questions. Translating these questions into research agendas is an important part of the overall prevention effort. Applied research is needed to improve strategies of subtyping and surveillance. Veterinary and agricultural research on the farm is needed to answer the questions about whether and how a pathogen such as *E. coli* O157:H7 persists in the bovine reservoir, to establish the size and dynamics of a reservoir for this organism in wild deer, and to look at potential routes of contamination connecting animal manure and lettuce fields. More research is needed regarding foods defined as sources in large outbreaks to develop better control strategies and better barriers to contamination and microbial growth and to understand the behavior of new pathogens in specific foods. Research is also needed to improve the diagnosis, clinical management, and treatment of severe foodborne infections and to improve our understanding of the pathogenesis of new and emerging pathogens. To assess and evaluate potential prevention strategies, applied research is needed into the costs and potential benefits of each or of combinations.

To prepare for the 21st century, we will enhance our public health food safety infrastructure by adding new surveillance and subtyping strategies and strengthening the ability of public health practitioners to investigate and respond quickly. We need to encourage the prudent use of antibiotics in animal and human medicine to limit antimicrobial resistance. We need to continue basic and applied research into the microbes that cause foodborne disease and into the mechanisms by which they contaminate our foods and cause outbreaks and sporadic cases. Better understanding of foodborne pathogens is the foundation for new approaches to disease prevention and control.

### References

1. Blake PA, Merson MH, Weaver RE, Hollis DG, Heublein PC. Disease caused by a marine vibrio: clinical characteristics and epidemiology. N Engl J Med 1979;300:1-5.
2. Riley LW, Remis RS, Helgerson SD, McGee HB, Wells JG, Davis BR, et al. Hemorrhagic colitis associated with a rare *Escherichia coli* serotype. N Engl J Med 1983;308:681.
3. Martin ML, Shipman LD, Wells JG, Potter ME, Hedberg K, Wachsmuth IK, et al. Isolation of *Escherichia coli* O157:H7 from dairy cattle associated with two cases of hemolytic uremic syndrome. Lancet 1986;2:1043.
4. Ortega YR, Sterling CR, Gilman RH, Cama VA, Diaz F. *Cyclospora* species—a new protozoan pathogen of humans. N Engl J Med 1993;328:1308-12.
5. Herwaldt BL, Ackers M-L, the Cyclospora Working Group. International outbreak of cyclosporiasis associated with imported raspberries. N Engl J Med. In press 1997.
6. Jackson LA, Wenger JD. Listeriosis: a foodborne disease. Infections in Medicine 1993;10:61-6.
7. Dekeyser PJ, Gossin-Detrain M, Butzler JP, Sternon J. Acute enteritis due to related *Vibrio*; first positive stool cultures. J Infect Dis 1972;125:390-2.
8. Tauxe RV. Epidemiology of *Campylobacter jejuni* infections in the United States and other industrialized nations. In: Nachamkin I, Blaser MJ, Tompkins L, editors. *Campylobacter jejuni*: current status and future trends, eds. Washington (DC): American Society of Microbiology, 1992. pp 9-19.
9. Tauxe RV, Vandepitte J, Wauters G, Martin SM, Goosens V, DeMol P, et al. *Yersinia enterocolitica* infections and pork: the missing link. Lancet 1987;1:1129-32.
10. World Health Organization. Worldwide spread of infections with *Yersinia enterocolitica*. WHO Chronicle 1976;30:494-6.
11. Rodrigue DC, Tauxe RV, Rowe B. International increase in *Salmonella enteritidis*: a new pandemic? Epidemiol Infect 1990;105:21-7.
12. Centers for Disease Control and Prevention. Multidrug-resistant *Salmonella* serotype Typhimurium—United States, 1996. MMWR Morb Mortal Wkly Rep 1997;46:308-10.
13. Endt HP, Ruijs GJ, van Klingeren B, Jansen WH, van der Reyden T, Mouton RP. Quinolone resistance in *Campylobacter* isolated from man and poultry following the introduction of fluoroquinolones in veterinary medicine. J Antimicrob Chemother 1991;27:199-208.
14. Lee LA, Puhr ND, Maloney K, Bean NH, Tauxe RV. Increase in antimicrobial-resistant *Salmonella* infections in the United States, 1989-1990. J Infect Dis 1994;170:128-34.
15. Cieslak PR, Noble SJ, Maxson DJ, Empey LC, Ravenholt O, Legarza G, et al. Hamburger-associated *Escherichia coli* O157:H7 in Las Vegas: a hidden epidemic. Am J Public Health 1997;87:176-80.
16. Humphrey TJ, Greenwood M, Gilbert RJ, Rowe B, Chapman PA. The survival of salmonellas in shell eggs cooked under simulated domestic conditions. Epidemiol Infect 1989;103:35-45.

17. Kirkland KB, Meriwether RA, Leiss JK, MacKenzie WR. Steaming oysters does not prevent Norwalk-like gastroenteritis. Public Health Rep 1996;111:527-30.
18. Holmberg SD, Feldman RA. New and newer enteric pathogens: stages in our knowledge. Am J Public Health 1984;74:205-7.
19. St. Louis ME, Morse DL, Potter ME, DeMelfi TM, Guzewich JJ, Tauxe RV, et al. The emergence of Grade A eggs as a major source of *Salmonella enteritidis* infections: implications for the control of salmonellosis. JAMA 1988;259:2103-7.
20. Mishu B, J Koehler, Lee LA, Rodrigue D, Brenner FH, Blake P, Tauxe RV. Outbreaks of *Salmonella enteritidis* infections in the United States, 1985-1991. J Infect Dis 1994;169:547-52.
21. Besser RE, Lett SM, Weber JT, Doyle MP, Barrett TJ, Wells JG, Griffin PM. An outbreak of diarrhea and hemolytic uremic syndrome from *Escherichia coli* O157:H7 in fresh-pressed apple cider. JAMA 1993;269:2217-20.
22. Keene WE, Sazie E, Kok J, Rice DH, Hancock DD, Balan VK, et al. An outbreak of *Escherichia coli* O157:H7 infections traced to jerky made from deer meat. JAMA 1997;277:1229-31.
23. Kohn MA, Farley TA, Ando T, Curtis M, Wilson SA, Jin Q, et al. An outbreak of Norwalk virus gastroenteritis associated with eating raw oysters: implications for maintaining safe oyster beds. JAMA 1995;273:466-71.
24. Tauxe R, Kruse H, Hedberg C, Potter M, Madden J, Wachsmuth K. Microbial hazards and emerging issues associated with produce; a preliminary report to the National Advisory Committee on Microbiologic Criteria for Foods. Journal of Food Protection. In press 1997.
25. Wang G, Zhao T, Doyle MP. Fate of enterohemorrhagic *Escherichia coli* O157:H7 in bovine feces. Appl Environ Microbiol 1996;62:2567-70.
26. Zhuang R-Y, Beuchat LR, Angulo FJ. Fate of Salmonella montevideo on and in raw tomatoes as affected by temperature and treatment with chlorine. Appl Environ Microbiol 1995;61:2127-31.
27. Rushing JW, Angulo FJ, Beuchat LR. Implementation of a HACCP program in a commercial fresh-market tomato packinghouse: a model for the industry. Dairy, Food and Environmental Sanitation 1996;16:549-53.
28. Tauxe RV, Hughes JM. International investigations of outbreaks of foodborne disease: public health responds to the globalization of food. BMJ 1996;313:1093-4.
29. Hennessey TW, Hedberg CW, Slutsker L, White KE, Besser-Wiek JM, Moen ME, et al. A national outbreak of *Salmonella enteritidis* infections from ice cream. N Engl J Med 1996;334:1281-6.
30. Passarro DJ, Reporter R, Mascola L, Kilman L, Malcolm GB, Rolka H, et al. Epidemic *Salmonella* Enteritidis infection in Los Angeles County, California: the predominance of phage type 4. West J Med 1996;165:126-30.
31. Centers for Disease Control and Prevention. Outbreaks of *Salmonella* serotype Enteritidis infection associated with consumption of raw shell eggs—United States, 1994-1995. MMWR Morb Mortal Wkly Rep 1996;45:737-42.
32. Centers for Disease Control and Prevention. Summary of notifiable diseases, United States, 1995. MMWR Morb Mortal Wkly Rep 1995;44(53).
33. Centers for Disease Control and Prevention. Proceedings of a national conference on salmonellosis, March 11-13, 1964. U.S. Public Health Service Publication No 1262. Washington (DC): U.S. Government Printing Office; 1965.
34. Bean NH, Morris SM, Bradford H. PHLIS: an electronic system for reporting public health data from remote sites. Am J Public Health 1992;82:1273-6.
35. Bean NH, Goulding JS, Lao C, Angulo FJ. Surveillance for foodborne-disease outbreaks—United States, 1988-1992. CDC Surveillance Summaries, October 25, 1996. MMWR Morb Mortal Wkly Rep 1996;45(SS-5).
36. Centers for Disease Control and Prevention. Foodborne Diseases Active Surveillance Network, 1996. MMWR Morb Mortal Wkly Rep 1997;46:258-61.
37. Centers for Disease Control and Prevention. Establishment of a national surveillance program for antimicrobial resistance in *Salmonella*. MMWR Morb Mortal Wkly Rep 1996;45:110-1.
38. Hutwagner LC, Maloney EK, Bean NH, Slutsker L, Martin SM. Using laboratory-based surveillance data for prevention: an algorithm for detecting Salmonella outbreaks. Emerg Infect Dis 1997;3:395-400.
39. Meriwether RA. Blueprint for a national public health surveillance system for the 21st Century. Journal of Public Health Management and Practice 1996;2:16-23.
40. Mahon BE, Pönkä A, Hall WN, Komatsu K, Dietrich SE, Siitonen A, et al. An international outbreak of *Salmonella* infections caused by alfalfa sprouts grown from contaminated seed. J Infect Dis 1997;175:876-82.
41. Bell BP, Goldoft M, Griffin PM, Davis MA, Gordon DC, Tarr PI, et al. A multistate outbreak of *Escherichia coli* O157:H7-associated bloody diarrhea and hemolytic uremic syndrome from hamburgers: the Washington experience. JAMA 1994;272:1349-53.
